# Apolipoprotein C1 (APOC1): A Novel Diagnostic and Prognostic Biomarker for Clear Cell Renal Cell Carcinoma

**DOI:** 10.3389/fonc.2020.01436

**Published:** 2020-08-20

**Authors:** Yankang Cui, Chenkui Miao, Chao Hou, Zengjun Wang, Bianjiang Liu

**Affiliations:** Department of Urology, The First Affiliated Hospital of Nanjing Medical University, Nanjing, China

**Keywords:** apolipoprotein C1 (APOC1), clear cell renal cell carcinoma (ccRCC), diagnosis, prognosis, biomarker

## Abstract

**Background:** Apolipoprotein C1 (APOC1) has been proved to play a critical role in gastric, breast, lung, and pancreatic cancer. However, the relationship between APOC1 and urinary tumors remains unclear. This study aimed to assess the diagnostic and prognostic value of APOC1 in urinary tumors.

**Methods:** We performed a pan analysis of APOC1 mRNA expression in urinary cancer using the Gene Expression Profiling Interactive Analysis (GEPIA) database. To further investigate the prognostic value of APOC1 expression in urinary cancers, the Kaplan-Meier plotter database was used. Furthermore, we collected the tumor and adjacent normal samples of 32 ccRCC patients to perform qRT-PCR and western blotting assays. A total of 72 cases with ccRCC were analyzed using tissue microarrays (TMAs).

**Results:** Our results based on Kaplan-Meier plotter database indicated that a high expression of APOC1 may lead to poor overall survival (OS, *p* = 0.0019) in patients with ccRCC. Furthermore, the cancer stages and tumor grade of ccRCC appeared to be strongly linked with APOC1 expression according to UALCAN database. Hence, we reached a preliminary conclusion that APOC1 may play a key role in the tumorigenesis and progression of ccRCC. Furthermore, the Kaplan-Meier survival curve analyses of 72 clinical patients indicated that high expression of APOC1 was associated with poor progression-free survival (PFS, *p* = 0.007) and OS (*p* = 0.022). In addition, univariate Cox regression analysis confirmed the significant relationship between APOC1 expression and survival (*p* = 0.038). The TMAs analysis in combination with the patients' clinicopathological features was also performed. The expression of APOC1 was found to be significantly correlated with the tumor size (*p* = 0.018) and histological grade (*p* = 0.016).

**Conclusions:** In conclusion, the findings of our study suggest that APOC1 may serve as a novel diagnostic and prognostic biomarker for ccRCC. Further evidence on the mechanism of APOC1 promoting tumor progression may transform it to a new therapeutic target for the treatment of ccRCC.

## Introduction

Kidney cancers account for ~2.2% of the global burden of all cancers, with more than 400,000 new diagnoses and 175,098 deaths worldwide in 2018 (1). Renal cell carcinoma (RCC) is the most common type, representing 85% of all kidney cancers (2). RCC consists of a family of carcinomas derived from the epithelium of renal tubules. The most frequent forms are clear cell renal cell carcinoma (ccRCC), papillary renal cell carcinoma, and chromophobe renal cell carcinoma. Approximately 80–90% of all RCCs are ccRCC, which is signified by the appearance of tumor cells with abundant clear cytoplasm (3). Patients with early stage ccRCC will benefit from timely surgical treatment, but for advanced tumors the 5-year survival rate is only 23% (2). Hence, it's of great urgency to improve our understanding of this disease and identify novel therapeutic targets with a better diagnostic and prognostic value.

Apolipoprotein C1 (APOC1), the smallest of all apolipoproteins (Mr = 6.6 kDa), is a member of the apolipoprotein C family and located at position 19q13.32. APOC1 is primarily expressed in the liver and activated when monocytes differentiate into macrophages (4). The encoded protein plays a central role in the metabolism of high-density lipoprotein (HDL) and very low-density lipoprotein (VLDL). This protein has also been shown to inhibit cholesteryl ester transfer protein in the plasma (5). In recent years, APOC1 was also reported to play significant roles in some biological processes, such as cholesterol catabolism, dendritic reorganization, and membrane remodeling (6, 7). APOC1 is associated with the progression of multiple diseases, including Alzheimer's disease, glomerulosclerosis, type 1 or type 2 diabetes, and diabetic nephropathy (8–11). Additionally, some studies revealed that APOC1 acts as an oncogene in the progression of some malignant tumors, including breast, pancreatic, colorectal, and lung cancer (11–15). However, the role of APOC1 in renal cancer has not been elucidated. The findings of our study revealed that APOC1 may act as an oncogene with novel prognostic and therapeutic target potential in ccRCC.

## Methods

### GEPIA Database Analysis

The transcription profiling of APOC1 gene expression in a variety of urinary cancers was performed using the Gene Expression Profiling Interactive Analysis (GEPIA) database (http://gepia.cancer-pku.cn/index.html). We used The Cancer Genome Atlas (TCGA) tumors vs. TCGA normal + The Genotype-Tissue Expression (GTEx) normal datasets to draw the expression box plots. The log2FC cutoff was set as 1, and p-value cutoff was 0.01. Genes with higher |log2FC| values and lower q values than preset thresholds are considered differentially expressed genes (15).

### Kaplan-Meier Plotter Database Analysis

The Kaplan Meier plotter (http://kmplot.com/analysis/) is used to assess the effect of 54 k genes on survival in 21 cancer types. The system includes gene chip and RNA-seq data-sources for the databases include Gene Expression Omnibus (GEO) and TCGA. The correlation between APOC1 mRNA expression and survival in kidney chromophobe (KICH), kidney renal clear cell carcinoma (KIRC), kidney renal papillary cell carcinoma (KIRP), prostate adenocarcinoma (PRAD), testicular germ cell tumors (TGCT), and bladder urothelial carcinoma (BLCA) was analyzed using the Kaplan-Meier plotter database. We split patients of all cancer stages to high and low APOC1 expression by auto select best cutoff. All possible cutoff values between the upper and lower quartiles are computed, and the best performing threshold is used as a cutoff. The log-rank p-value and hazard ratio with 95% confidence interval were also calculated. The follow-up threshold contained all survival time.

### UALCAN Database Analysis

UALCAN (http://ualcan.path.uab.edu/index.html) is an interactive web portal for in-depth analysis of TCGA gene expression data (16). Here, we used UALCAN to investigate the potential relationship between the APOC1 expression level and tumor malignancy including cancer stage and tumor grade.

### Sample Collection

The ccRCC tumor and normal tissues were acquired from patients who were diagnosed with ccRCC and underwent surgery at The First Affiliated Hospital of Nanjing Medical University between 2010 and 2018. Patients who were diagnosed with ccRCC by pathology were included, and those who had any medical history of other neoplasms were excluded. Finally, a total of 72 pairs of tissues were included in the cohort; 1 patient had metastasis by the time of surgery. We collected the clinical data and pathological features of these patients who were included in these tissue microarrays (TMAs). The deadline date of follow-up was July 2018. Samples for RNA and protein extraction were freshly frozen in liquid nitrogen and stored at −80°C. Samples for immunohistochemical analysis were formalin fixed. The study design and protocol was approved by the ethics committee of The First Affiliated Hospital of Nanjing Medical University. All patients included in this study provided informed consent.

### Cell Culture and Treatment

Human renal cancer cell lines (786-O, 769-P, CAKI-1, CAKI-2, and ACHN) and human renal tubular epithelial cells (HK-2) were purchased from the Chinese Academy of Sciences (Shanghai, China). All cell lines were cultured at 37°C with 5% CO2. CAKI-1 and CAKI-2 were cultured in McCoy's 5A Medium (Gibco, Waltham, MA, USA); 786-O and 769-P were cultured in RPMI-1640 medium (Gibco, Grand Island, NY, USA). HK-2 was cultured in DMEM. The medium was supplemented with 10% fetal bovine serum (FBS) (Gibco, Grand Island, NY, USA).

### RNA Extraction, Reverse Transcription, and Quantitative RT-PCR

Total RNA was extracted from renal tissues and cell lines using TRIzol reagent (Invitrogen, Carlsbad, CA, USA), following the manufacturer's protocol. The total RNA was reverse transcribed into complementary DNA (cDNA) using HiScript II (Vazyme, Shanghai, China). qRT-PCR was performed using SYBR Green I (Vazyme, Shanghai, China) on ABI 7900 system (Applied Biosystems, Carlsbad, CA, USA) and the primers for APOC1 were as follows: forward(F), 5′-AGGACAAGGCTCGGGAACTCAT-3′, and reverse(R), 5′-GATGTCACCCTTCAGGTCCTCA-3′. The primers for β-actin were as follows: (F), 5′-GAAGATCAAGATCATTGCTCCT-3′, and (R), 5′-TACTCCTGCTTGCTGATCCA-3′.

### Western Blotting

Cell lines and renal tissues were lysed using RIPA Lysis Buffer (Beyotime biotechnology, Shanghai, China), and proteins were harvested and quantified using the bicinchoninic acid (BCA) kit (Beyotime biotechnology, Shanghai, China). Proteins were separated on a 15% gel using sodium dodecyl sulfate (SDS)-PAGE and transferred onto polyvinylidene fluoride (PVDF) membranes (Sigma-Aldrich, St. Louis, MO, USA). The membranes were blocked in Tris-buffered saline (TBS) containing 5% non-fat milk for 2 h. After incubation with an anti-APOC1 antibody (1:1,000, ab198288, Abcam), and an anti-GAPDH antibody (1:2,500, ab9485, Abcam) overnight at 4°C, the membranes were washed three times with TBS-T (TBS containing 0.1% Tween-20). Subsequently, the membranes were incubated in a secondary antibody solution at room temperature for 2 h. After washes, the signals were detected using the chemiluminescence system and analyzed with Image Lab Software.

### Immunohistochemistry (IHC)

IHC staining and evaluation of it was performed as in previously described methods (17). In brief, the protein expression of APOC1 in serial ccRCC tumor tissues from TMAs was detected by anti-APOC1 antibody (1:400, ab198288, Abcam) and a secondary antibody (1:5,000, L3012-2, SAB). Immunoreactive score of Remmele and Stegner (IRS) system was used to determine the protein expression level. From previous methods, a final score >1 was considered a high APOC1 expression; otherwise, it was considered as low APOC1 expression.

### Statistical Analysis

Chi-square test or Fisher's exact test was used to assess the associations between the protein expression level and clinicopathological factors. Kaplan-Meier curve and log-rank test were used to compare the progression free survival (PFS; progression-free survival was defined as the time between the diagnosis and the first unequivocal clinical or radiological sign of disease progress) and overall survival (OS; overall survival was defined as the time from randomization until death from any cause) in the study cohort. Additionally, univariate Cox regression analysis was performed. The Student's *t*-test was used to compare differences between two or three groups, and *P* < 0.05 was considered statistically significant. The analyses were performed using the SPSS 16.0 software and GraphPad Prism 7.0.

## Results

### APOC1 Is Increased in Urinary Tumors and Correlates With the Prognosis of ccRCC in Patients Based on the Public Databases

**Figure 1:**
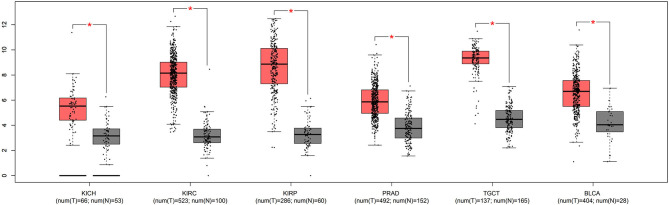
The mRNA expression of APOC1 was significantly increased in urinary tumor tissues, compared to that in normal tissues (*p <0.05).

We first investigated whether the mRNA expression of APOC1 was altered in urinary tumors. The results from the GEPIA database revealed that the APOC1 level was significantly increased in tumor tissues compared to that in normal tissues (Figure 1).

**Figure 2:**
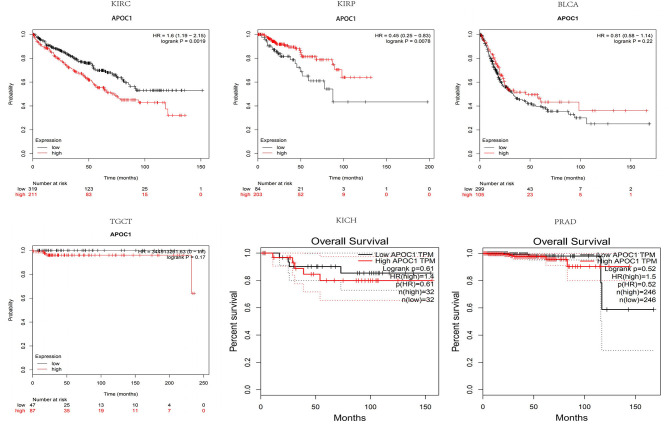
Higher expression of APOC1 was significantly associated with shorter overall survival in KIRC (*p* = 0.0019). However, no such association was observed in KIRP (*p* = 0.0078), despite the high APOC1 levels. Additionally, high APOC1 had no significant association with the overall survival in other urinary tumors, including BLCA (*p* = 0.22), TGCT (*p* = 0.17), KICH (*p* = 0.61), and PRAD (*p* = 0.52).

To further explore the APOC1 expression pattern and its prognostic significance in urinary tumors, we used Kaplan-Meier plotter database to draw survival curves. As shown in Figure 2, high expression of APOC1 was significantly associated with shorter OS in ccRCC (*p* = 0.0019). However, longer overall survival was found in KIRP (*p* = 0.0078) with high level of APOC1. Additionally, high APOC1 had no statistical difference on the overall survival of other urinary tumors, including BLCA (*p* = 0.22), TGCT (*p* = 0.17), KICH (*p* = 0.61), and PRAD (*p* = 0.52).

### APOC1 Promotes Tumor Progression in ccRCC

**Figure 3:**
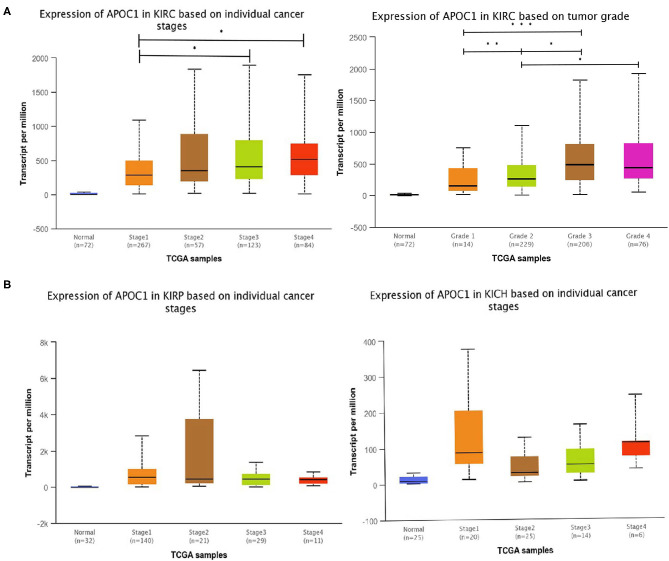
**(A)** The transcript level of APOC1 in different stages of kidney cancers. The expression of APOC1 increased with the development of KIRC, but made no sense for KIRP and KICH. **(B)** Similarly, the significantly difference in APOC1 expression was also found in ccRCC tumor grades.

Given that APOC1 was upregulated in ccRCC tissues and its high expression led to shorter OS, we further investigated the role of APOC1 in the tumor progression of ccRCC based on the cancer stage and tumor grade. According to the analysis of UALCAN database (Figure 3A), we found that the expression of APOC1 increased with the development of KIRC, but no such observation was made in case of KIRP and KICH. Furthermore, higher expression of APOC1 was observed in higher tumor grade of KIRC (Figure 3B). Therefore, we speculated that APOC1 may act as an oncogene in ccRCC to promote tumor progression.

### Aberrant Expression of APOC1 in RCC Tumor Specimens and Cell Lines

**Figure 4:**
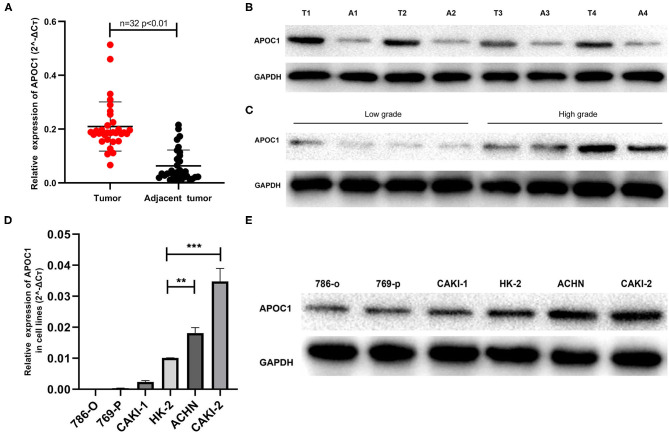
**(A)** PCR results of the 32 ccRCC tumor tissues. The mRNA expression of APOC1 in tumor tissues was significantly higher than that in adjacent tissues (*p* < 0.01) **(B)** the APOC1 protein level was significantly higher in tumor tissues than that in adjacent tissues. **(C)** The western blot analysis of eight ccRCC patients with different tumor grades revealed higher protein expression of APOC1 in patients with higher tumor grade. **(D,E)** The mRNA and protein expression level of APOC1 was highest in CAKI-2. In addition to CAKI-2 and ACHN, all the other cell lines showed lower APOC1 expression than HK-2, which is considered as a normal tissue cell line (**p < 0.01, ***p < 0.001).

To verify the above results, we subsequently performed qRT-PCR and western blot for the ccRCC tumor samples. A total of 32 ccRCC tumor tissues and adjacent normal tissues (normal tissues adjacent to the tumor volume) were collected. The PCR result is shown in Figure 4A. The transcriptional expression of APOC1 in tumor tissues was significantly higher than that in the adjacent tissues (*p* < 0.01). Similarly, the APOC1 protein level was also found to be higher in tumor tissues (Figure 4B).

Subsequently, we investigated whether APOC1 was expressed in renal cancer cell lines (786-O, 769-P, CAKI-1, CAKI-2, and ACHN) and normal cell line HK-2. As displayed in Figures 4D,E, the mRNA and protein expression levels of APOC1 were highest in CAKI-2. In addition to CAKI-2 and ACHN, all the other cell lines showed a lower expression of APOC1 than HK-2.

The difference in the expression of APOC1 in the progression of ccRCC was also verified in this study. We collected eight ccRCC tumor tissue samples, half of which were with high Fuhrman grade (grade: 3–4) and half were with low Fuhrman grade (grade: 1–2), based on postoperative pathology. The western blot analysis revealed that higher protein expression of APOC1 could be found in patients of ccRCC with higher tumor grade (Figure 4C). These results were consistent with our current findings.

### Expression Pattern of APOC1 in Clinical RCC Cohorts and Its Prognostic Validation

**Figure 5:**
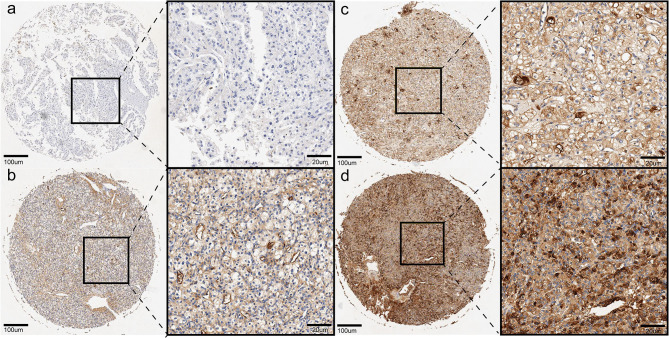
Representative pictures of ccRCC in tissue microarray by IHC, **(a)** (negative), **(b)** (weak brown), **(c)** (moderate brown), **(d)** (strong brown).

IHC staining assay was performed using TMAs tissues of our clinical cohort. As shown in Figure 5, a total of 72 ccRCC patients were classified into the low APOC1 expression group (IRS ≤ 1) and high APOC1 expression group (IRS>1) based on the IHC staining score. Finally, 45 of the 72 (62.5%) samples showed high expression of APOC1, while 27 (37.5%) of tissues with relatively low APOC1 level. The association between the APOC1 expression and clinicopathological characteristics of patients were summarized in Table 1. We found that high expression of APOC1 was significantly associated with the larger tumor size (*p* = 0.018) and advanced histological grade (*p* = 0.016). However, APOC1 expression status had no significant difference in the age, gender, and TNM stage of ccRCC patients.

**Table 1:** Correlations between the expression of APOC1 and clinicopathological features in 72 ccRCC patients.

**Characteristics**	**Case**	**APOC1 expression**		***p***
		**Low**	**High**	
All cases	72	27	45	
Age(years)				0.219
<60	48	16	32	
≥60	24	11	13	
Gender				0.452
Male	46	18	28	
Female	26	9	17	
TNM stage				0.393
T1	61	22	39	
T2–T4	11	5	6	
Tumor size(cm)				0.018^*^
≤ 4	38	19	19	
>4	34	8	26	
Histological grade				0.016^*^
I and II	52	15	37	
III and IV	20	12	8	

* *P < 0.05*.

**Figure 6:**
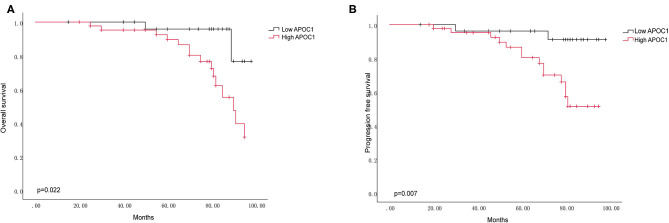
The APOC1 high expression group was associated with decreased overall survival **(A)** and progression-free survival **(B)** in 72 ccRCC patients. Log-rank *p* value was 0.022 for OS, and 0.007 for PFS.

In addition, we performed the outcome analysis of our clinical cohort to validate the prognostic significance of APOC1 expression in ccRCC. Kaplan-Meier curves revealed that the patients with high APOC1 expression had a shorter OS and PFS (*p* = 0.022 for OS and *p* = 0.007 for PFS, Figures 6A,B). Finally, univariate Cox regression analysis for the survival analysis was conducted to explore the significant value of APOC1 in prognosis (Table 2; HR, 0.209; 95% CI, 0.048–0.916 [*p* = 0.038]).

**Table 2:** Univariate Cox regression for OS.

**Variable**	**HR (95% CI)**	***P***
APOC1 expression	0.209 (0.048–0.916)	0.038^*^

*HR (95% CI), hazard ratio, with 95% confidence interval; OS, overall survival*;

* *P < 0.05*.

## Discussion

In this study, we first investigated the APOC1 expression profile in urinary tumors and its relevant prognostic value in ccRCC. We compared the expression level of APOC1 in the urinary tumor tissues and normal tissues using databases and found a general higher expression in tumors as compared to that in normal tissues. Besides, high expression of APOC1 made a significant difference in the overall survival of patients with ccRCC and KIRP. We further investigated the role of APOC1 in the tumor progression of ccRCC and KIRP. Our observation found higher APOC1 expression in advanced tumor malignancy characterized by tumor grade and cancer stage of ccRCC. To verify the results from the analysis of databases, we collected tissue samples from patients with ccRCC to perform subsequent validation using qRT-PCR, western blotting, and IHC staining assays. The obtained results were consistent with our previous findings using the databases. Moreover, by analyzing the associations between APOC1 expression and the clinicopathological characteristics of patients with ccRCC, we confirmed the correlation between APOC1 expression and tumor size, which reflects the tumor's aggressiveness.

It is known that ccRCC is highly aggressive and distant metastasis often occurs during advanced tumor stage. About 30% of all patients with ccRCC have metastases at the time of diagnosis, and another 30–40% will develop metastases at a later stage (18). Recently, despite the development of molecular targeted therapies, ccRCC patients' treatment is still challenging once metastasis is manifested, leading to a 5-year survival of only 23% (2, 19). Hence, it's of pressing requirement indeed to identify effective targets to diagnose and intervene ccRCC at an early stage.

The ccRCC cells have the aggregation characteristics of cholesterol, cholesterol ester, and other lipids (20), suggesting that the content of cholesterol and cholesterol ester in ccRCC tissues is higher than that in normal kidney tissue (21). Cholesterol has been demonstrated to slightly promote the ccRCC cell proliferation, but it significantly increases the capacities of invasion and migration by regulating the KLF5/miR-27a/FBXW7 axis (22). APOC1 is present in chylomicrons, VLDL, and HDL. APOC1 acts as an exchangeable apolipoprotein between these lipoprotein classes with an important role in lipid transport, metabolism, and homeostasis (23). *In vivo*, the overexpression of human APOC1 in mice led to hyperlipidemia owing to the reduced uptake of VLDL and post-lipolysis particles by inhibiting the binding of VLDL to VLDLR (24). This could be one of the contributing factors in the high APOC1 expression leading to poor clinical outcomes for patients with ccRCC. Therefore, targeting APOC1 to regulate cholesterol metabolism may be a novel treatment approach.

Recently, the relationship between APOC1 and malignant tumors has been highlighted. APOC1 was demonstrated to promote cell proliferation in prostate cancer cells *in vitro* (25). In gastric cancer (GC), APOC1 revealed the value of diagnosing and prognosing for GC (17). In colorectal cancer, APOC1 played its proliferative activity by MAPK signaling (13), and in pancreatic cells it was found to inhibit apoptosis (12). Herein, our findings for the first time revealed that APOC1 could be considered as a potential diagnostic and prognostic biomarker for ccRCC. However, the limitation that cannot be ignored is the small sample scales in our study, which might weaken the statistical association between APOC1 expression and ccRCC progression. Nevertheless, analysis of ccRCC patients relying on TCGA datasets was also performed and further confirmed our results. Further research should be undertaken to uncover the potential mechanism of APOC1 promoting ccRCC tumor progression by regulating cholesterol metabolism. Better understanding of this may give a new hope for the treatment of advanced ccRCC patients.

## Conclusions

In summary, this is the first study to investigate the role of APOC1 in ccRCC. APOC1 expression was much higher in ccRCC tumor tissues, and high expression of APOC1 correlated with a shorter OS, PFS, and poor clinical characters, including cancer stage, tumor grade, and tumor size. We speculate that APOC1 might act as a tumor promoter by regulating the cholesterol metabolism. However, further research should be carried out to reveal the underlying mechanism. Cumulatively, APOC1 may be a promising biomarker to diagnose ccRCC and predict prognostic outcomes in ccRCC patients.

## Data Availability Statement

The datasets generated for this study are available on request to the corresponding author.

## Ethics Statement

The studies involving human participants were reviewed and approved by Medical ethics committee of The First Affiliated Hospital of Nanjing Medical University. The patients/participants provided their written informed consent to participate in this study.

## Author Contributions

YC and CM designed the study. YC collected the data and edited the manuscript. CH analyzed the data. CM and ZW sourced the literature. BL acquired the funding and supervised the whole study. All authors contributed to the article and approved the submitted version.

## Conflict of Interest

The authors declare that the research was conducted in the absence of any commercial or financial relationships that could be construed as a potential conflict of interest.
